# Intersectoral strategies between health and education for preventing adolescent pregnancy in Chile: Findings from a qualitative study

**DOI:** 10.1111/hex.12840

**Published:** 2018-10-05

**Authors:** Alexandra Obach, Michelle Sadler, Báltica Cabieses

**Affiliations:** ^1^ Social Studies in Health Research Programme Facultad de Medicina Clínica Alemana Universidad del Desarrollo Santiago Chile; ^2^ Department of History and Social Sciences Faculty of Liberal Arts Universidad Adolfo Ibáñez Santiago Chile; ^3^ Medical Anthropology Research Center Universitat Rovira i Virgili Tarragona Spain; ^4^ Department of Health Sciences University of York York UK

**Keywords:** adolescent health, adolescent health services, intersectoral collaboration, pregnancy in adolescence, qualitative research, reproductive health, sexual health

## Abstract

**Background:**

In Chile, despite its steady decrease overall, adolescent pregnancy is concentrated in the most vulnerable population. Efforts in intersectoral collaboration between health and education to address the problem are being developed, but they have not been assessed.

**Objective:**

To describe intersectoral strategies between health and education to address adolescent sexual and reproductive health, prevent adolescent pregnancy, and to explore adolescents’ and health professionals’ perceptions regarding those strategies.

**Design:**

A qualitative ethnographic study was carried out in five municipalities in the Metropolitan Region of Chile. A sample of five key informants, 23 health professionals and 50 adolescents participated in a total of 38 semi‐structured interviews and five discussion groups.

**Results:**

Two intersectoral strategies to respond to adolescents’ sexual and reproductive health needs were identified: (a) the “in‐and‐out” strategy, where health professionals provide health care mostly in health centres and carry out specific actions in schools and (b) the school‐based strategy in which health professionals carry out continuous actions in schools as part of the curriculum. The second is perceived as responding better to adolescents’ needs in sexual and reproductive health issues and in preventing adolescent pregnancy.

**Discussion:**

The school‐based strategy, with the constant presence of health professionals and lack of bureaucratic procedures, facilitates adolescents to access sexual and reproductive health care. This strategy enables sexual and reproductive health to be understood as an integral dimension of adolescents’ lives, and it reinforces a holistic idea of health in which it is approached as a whole.

## 1 INTRODUCTION

Adolescence is a period of life between the ages of 10 and 19 where particular health risks are faced, especially related to reproduction and sexuality.^1^ Globally, adolescents are considered a priority group due to their vulnerability and the unfavourable conditions in which many of them live and develop. Factors such as low educational level, high rates of unplanned pregnancy and other social determinants disfavour and socially exclude them, particularly in developing countries.^1^, ^2^ While adolescent pregnancy figures are declining around the world, they still account for 11% of all births worldwide^2^ and are a major international public health concern because they have profound consequences for individuals, the community and society as a whole.^3^, ^4^, ^5^, ^6^, ^7^ They cause a set of negative consequences, especially for women, perpetuating poverty, social inequality and gender inequities.^8^, ^9^

Worldwide, the adolescent fertility rate for 2010‐2015 was of 51 births per 1000 girls aged 15‐19.^10^ Latin America and the Caribbean were above the world average and presented the second highest adolescent fertility rate in the world, estimated in 67 births per 1000 girls for 2010‐2015, only after Sub‐Saharan Africa.^10^ During the same period, Chile presented an adolescent fertility rate of 49.3 per 1000, one of the lowest rates of the Region.^8^ Nonetheless, adolescent pregnancy continues to reflect significant social inequality, being concentrated in the lower socio‐economic strata.^4^, ^8^, ^11^ In 2013, Chilean girls aged 15‐19 from the lowest income quintile of the population were up to six times more likely to become mothers than those from the highest income quintile.^8^

Since 2008, the Chilean Health Ministry has implemented various strategies to improve the health and development of adolescents. In 2009, the first 54 Youth‐Friendly Health Services (YFHS) were implemented within primary health centres, to respond to the need for differentiated health care for adolescents aged 10‐19.^12^ The work by the YFHS was reinforced by the creation of the National Adolescent Health Programme in 2012.^13^ By 2016, the number of YFHS centres in Chile had increased to 243.^14^ Like similar services elsewhere in the world, Chile's YFHS focus on ensuring health service availability and making their provision adolescent‐friendly, that is: accessible, acceptable, equitable, appropriate and effective for this population.^1^ They take a promotional‐preventive approach to health with a special emphasis on sexual and reproductive health and prevention of adolescent pregnancy. As well, they have implemented differentiated service hours for adolescents to make visits compatible with school schedules and have adapted protocols and infrastructure for making adolescent's access as expeditious and private as possible.^12^

Since the creation of the YFHS, a number of studies have been carried out in Chile to understand the barriers teenagers face in accessing sexual and reproductive health care. Results have shown that some adolescents are unaware of the existence of YFHS, while those who are aware fear a lack of privacy and confidentiality and believe that being admitted for care will entail excessive bureaucracy.^15^, ^16^ The health professionals working with adolescents report negative prejudices regarding adolescents’ autonomy and that they lack the skills for working with this age group on topics of gender and sexual and reproductive rights.^15^ A recent qualitative study in a municipality of Santiago, the capital city, reinforces the idea that health centres are perceived by adolescents as distant and bureaucratic, with little awareness of adolescents and their needs in terms of sexual and reproductive health issues.^17^ Chile's predominant framework of sexual and reproductive health has been characterized as (a) being strongly heteronormative and biologicist, (b) reinforcing a risk approach, (c) being focused mainly on girls, thus excluding boys and young men as recipients of sexual and reproductive health care and (d) failing to promote adolescents’ participation and rights.^18^, ^19^, ^20^ These findings are similar to those from research in other regions of the world.^21^

A promising strategy for overcoming these barriers has been an intersectoral approach to adolescent health that includes the health and education sectors. The school environment is considered strategic for promoting programmes about adolescent sexual and reproductive health because of its key role in the social development of children and young people.^22^, ^23^ In Chile, schools constitute an ideal scenario for expanding health actions because the country has 12 years of mandatory school education with low dropout rates.^24^ Following international guidelines,^25^ the YFHS protocols have been progressively expanded to include joint actions with the educational sector. However, no detailed model for collaboration between the two sectors has been specified, so it is locally determined^26^ and varies widely in practice.

Internationally, some standalone adolescent health centres and out‐of‐facility settings, such as school‐based healthcare delivery, have been developing for decades.^22^, ^23^, ^27^, ^28^, ^29^ However, in Chile and Latin America, such initiatives are recent and incipient, and little is known about them.^10^ In this context, a qualitative research project was carried out, which aimed to describe the provision of sexual and reproductive health services focussed on preventing adolescent pregnancies in five YFHS in Chile's Metropolitan Region. The study was carried out in the public health sector, considering that young people from the lower socio‐economic strata should be addressed as a priority group in policy design, which should incorporate their specific needs and especially reduce the gaps in their access to sexual and reproductive health services.

The objectives of this paper are to describe intersectoral strategies between health and education in Chile to address adolescents’ sexual and reproductive health and prevent adolescent pregnancy, and to explore adolescents’ and health professionals’ perceptions regarding those strategies. The paper seeks to contribute to the international discussion about strengthening intersectoral strategies that promote the sexual and reproductive rights of adolescents, and the prevention of adolescent pregnancy.

## 2 METHODS

The study had a qualitative design, seeking to understand how people give meaning to their social environment and how they interpret it.^30^ It followed an ethnographic method, which observes and describes the cultural practices specific to particular social groups in their natural settings.^31^ This methodological design was chosen, since it emphasizes the importance of context to understanding events and meanings.^32^ A detailed description of the methodology has been published elsewhere.^33^

Fieldwork was carried out during 2016 in five municipalities (Chile's smallest administrative subdivisions) of the Metropolitan Region, under the administration of the West Metropolitan Health Service. The choice of health service and municipalities was based on those presenting some of the highest rates of adolescent pregnancy in the region: in 2015, the mothers of 12.4% of newborns in these five municipalities were 15‐19 years old; whereas, the regional average was of 8.8% newborns for the same age group.^34^

In each municipality, ethnographic research was carried out within one selected YFHS. Three of the YFHS studied were located in primary health centres, following the Health Ministry's guidelines, as spaces enabled exclusively for adolescents with schedules compatible with school hours (usually 4 pm‐8 pm, once or twice a week). One was a standalone adolescent health centre that works independently from the primary health centres of its municipality, focusing only on adolescent health. In the fifth municipality, fieldwork was carried out in two secondary schools. In this municipality, YFHS in secondary schools have been implemented in parallel to those located in health centres.

The following research techniques were used: (a) participant observation of the dynamics of the YFHS, especially interactions between adolescents and health professionals; (b) semi‐structured interviews with key informants (head teachers, youth organization representatives and managers of health services), health professionals working directly with adolescents, and adolescents; (c) discussion groups with health professionals, and with adolescents. Table 1 shows the detail of the semi‐structured interviews and discussion groups carried out. Both semi‐structured interviews and discussion groups were used because they achieve different objectives: the first enables more detailed, in‐depth information to be obtained regarding the study questions at an individual level, while the second explores points of encounter and disagreement at group level.^31^ For both techniques, an interview guide was used to explore the following areas regarding sexual and reproductive health: adolescents’ needs and sexual practices, interactions between health professionals and adolescents in different settings, perceptions about adolescent parenthood and strategies for preventing adolescent pregnancy. In addition to exploring these specific topics, other topics could emerge freely. Interviews and groups were conducted by AO, MS and a research assistant. Field diaries were kept to register observations, and interviews and groups were audio‐recorded.

**Table 1:** Interviews and discussion groups

	Semi‐structured interviews	Discussion groups: 2 with health professionals, 3 with adolescents	Total participants
	No participants	No participants	
Key informants	5	–	5
Health professionals	10	13	23
Adolescents	23	27	50
Total	38	40	78

The project and protocol for obtaining informed consent were approved by the West Metropolitan Health Service's Ethics Committee. All the participants aged over 18 signed an informed consent document. Those under 17 signed an assent document, and their parents/guardians signed a consent document. Of all the participants invited for the study, five adolescents were excluded because of the lack of parental consent.

Key informants and health professionals were recruited by being invited directly within the study settings. The health professionals included in the sample were those working with adolescents in the YFHS selected for fieldwork. They were mainly midwives, followed by social workers, and in smaller numbers psychologists, nutritionists, health technicians and one nurse. Adolescents were recruited through three mechanisms: (a) being contacted through health professionals and teachers; (b) directly by the research team in waiting rooms of health centres (c) and through snowball sampling, in which the first participants recruited adolescents they knew. These adolescents were aged 15‐19, with no exclusion criteria. The sample was intended to include pregnant young women and men with pregnant partners, young women and men who had already been mothers/fathers and others who had not. They had diverse ethnic backgrounds, identified with different sexual orientations, and may or may not have sought sexual and reproductive health services in the YFHS (this was in order to contrast opinions and experiences of users and non‐users of such services). Table 2 displays the characteristics of the health professionals and adolescents who participated in the study.

**Table 2:** Characteristics of health professionals and adolescents who participated in semi‐structured interviews and discussion groups (excluding key informants)

Health professionals, N = 23	N	%^a^
Sex^b^		
Female	21	91.3
Male	2	8.7
Profession		
Midwife	9	39.1
Social worker	6	26
Psychologist	3	13
Nutritionist	2	8.7
Technician	2	8.7
Nurse	1	4.3
Age		
23‐30	11	47.8
31‐40	8	34.8
41 or more	4	17.4
Years working with adolescents		
0‐1	4	17.4
1‐3	5	21.7
3‐6	7	30.4
6 or more	7	30.4
Adolescents, N = 50		
Sex		
Female	25	50
Male	25	50
Age		
15‐16	17	34
17‐19	33	66
Nationality/ethnicity		
Chilean	41	82
Chilean‐Mapuche	5	10
Non‐Chilean Nationality (Peruvian, Ecuadorian, Colombian)	4	8
Sexual orientation		
Heterosexual	46	92
Homosexual	3	6
Bisexual	1	2
Use of youth‐friendly health spaces^c^		
User	30	60
Non‐user	20	40
Parenthood		
No children	44	88
1 child	4	8
Pregnant	2	4

a Numbers may not add exactly to 100% because they were rounded to the first decimal.

b Most health professionals who work in sexual and reproductive health care at the primary level are women.

c As the field work was focused on YFHS, the % of adolescents who were current users of such services was higher than those who do not seek health services in those spaces.

Field notes from observations and audio recordings from interviews and discussion groups were transcribed verbatim, and analysed using thematic analysis, a qualitative method that enables thematic patterns to be identified and analysed from the collected data^31^ with the support of NVivo software. The interviews were conducted in Spanish. The verbatim quotations were translated by a native English‐speaker translator and checked by the authors to verify the translations had captured their original meaning.

The following strategies were used to assess the rigour of the study (credibility, dependability and confirmability)^30^: (a) prolonged engagement and persistent observation in the field to gain a full understanding of the phenomena being investigated;^35^ (b) triangulation of responses from several techniques (semi‐structured interviews, discussion groups and participant observation) and triangulation of participants (key informants, health professionals, adolescents); (c) audit trail, by maintaining a field diary to record the ideas and experiences of the research team; (d) and two member‐checking meetings, one with health professionals and one with adolescents.^36^ All those who had participated in interviews and discussion groups were invited to these meetings. A low turnout was expected; nonetheless, 18 health professionals and 41 adolescents attended the meetings, where the main findings of the research were presented. Participants were invited to give their opinions, which were useful for providing missing information and deepening certain topics.^37^

## 3 RESULTS

### 3.1 Adolescents’ sexual and reproductive health needs

The main need reported by adolescents and confirmed by the health professionals is access to quality information and counselling, on topics ranging from physiological processes, such as their reproductive cycles, preventing pregnancy and sexually transmitted diseases, to the affective dimensions of sexuality and parenthood. One midwife said: “They are not clear about things as basic as why they start menstruating, their anatomy.”

The adolescents interviewed (YFHS users and non‐users) said that their main sources of information on sexuality issues are the internet, social networks and peer groups, but, as a key informant alerted: “…on the internet the problem isn't that information is missing, it's that there's a lot. And… knowing how to discriminate what's true from what's false, that's the problem.” Irrespective of this, many adolescents said that the internet is their main information source, since they feel that none of society's formal institutions (family, education, health services) provide them with adequate tools. A 16‐year‐old male non‐user of YFHS stated: “Almost everything I've learned, I learned from friends… in the street, but never with my family or at school, so I learned everything outside.” Adolescents who had accessed sexual and reproductive health services in YFHS acknowledged the information given by health professionals to be more accurate and complete that that from other sources, but it focuses primarily on biological processes and not on the emotional aspects of sexuality.

Regarding the family, most of the adolescents felt that their parents consider sexuality as a taboo topic, not to be discussed. As one 17‐year‐old male user of YFHS commented: “Most families are embarrassed or nervous to talk about it with their children”. Young interviewees declared that schools teach basic sexual education that does not satisfy their needs. A 15‐year‐old female non‐user of YFHS stated: “They never spoke clearly, never said ‘it′s when the woman and the man’ (…) never did so, there was no firm basis… I had friends who were already losing their virginity and didn't know anything, didn't know what the birth control pill was, they didn't know what condoms were.”

The adolescents acknowledged that this lack of quality information leads to unwanted pregnancies, which could be avoided with adequate sexual and reproductive health education. Knowing how to effectively prevent pregnancy appears to be an urgent need. A 17‐year‐old female user of YFHS declared: “I don't think we look for it [pregnancy], I think it's just a lack of education, where one thing leads to another. If you don't have the basic knowledge of what it is to get pregnant and you cannot prevent a pregnancy, you're going to have sex anyway… and that is the concern of us as teenagers.”

The adolescents also recognized a growing acceptance of different sexual identities; for example, some of them spoke about themselves as heterocurious. A 16‐year‐old male non‐user of YFHS, defined it as follows: “Totally, heterocurious (…) I believe it′s how we name a kind of unconfirmed bisexuality. One says: “I like the opposite sex but occasionally I've tried, I've tried something there, I can see what there is.” This was confirmed by health professionals; as one social worker stated: “The needs of adolescents have changed; ten years ago sexual diversity wasn't an issue (…). Now we have to give space to the needs of the trans, the lesbian. I feel they don't have an adequate space in the health system.” While recognizing the growing visibility of the issue, both adolescents and health workers said the health system fails to provide adequate information and guidance on this topic, as one midwife confirmed: “They [adolescents] lack information or they don't speak much about the topic because they don't know how to take care of themselves in relations that are homosexual (…) and we lack elements to guide them properly.”

The need for quality information on sexual and reproductive health is common to all adolescents, although the users of YFHS reported a higher understanding of physiological processes than those who had not accessed them. In most adolescents who were sexually active, the desire to start or continue using contraceptive methods was fundamental. Confidentiality was also mentioned as an important need for all adolescents, and, while the current health regulations guarantee confidentiality in health care for adolescents over 14, some said that they could only access health care if a parent were present. For instance, one 15‐year‐old female user of YFHS said: “They [health professionals] said I could only go alone if I was over 18; if not, with my mother.”

### 3.2 Approaches to adolescent sexual and reproductive health

Key informants and health professionals agreed that the topic of adolescent health has been on Chile's national agenda in recent years. The greatest perceived progress is related to an increase in (a) health professionals’ training and awareness of adolescence, gender and masculinities; (b) coverage, because of the increased number of YFHS, which entail fewer bureaucratic obstacles, differentiated schedules and adequate physical spaces for attending young people. Despite this perceived progress, they consider that the health sector still lacks effective strategies for approaching adolescents. The development of intersectoral strategies, especially with education, therefore becomes crucial to reaching adolescents wherever they are, rather than waiting for them to approach the health centres. Although the YFHS Programme explicitly stipulates that health professionals in charge of adolescent care must do extramural activities in schools, the type of activities and the number of hours allocated are not specified, and they differ widely between contexts.

The five YFHS studied present important differences in the way they approach intersectoral work with schools and in the way they address adolescents’ sexual and reproductive health needs. Thus, the study included two different strategies for linking the health and educational sectors, as shown in Table 3.

**Table 3:** Strategies for intersectoral collaboration between health and education

	“In‐and‐out” strategy	School‐based strategy
Location of YFHS	YFHS in primary health centres and as a standalone centre.	YFHS simultaneously in primary health centres and schools in the same municipality.
Actions to link health and education sectors	Health professionals from the YFHS provide health care to adolescents preferably in the primary health centres and carry out specific actions in schools, such as: health controls...: Health controls, counselling actions (health promotion, prevention of unplanned pregnancy, sexually transmitted diseases, HIV/AIDS and other issues associated with adolescent sexual behaviours), workshops on sexual education.	Health professionals carry out continuous actions in schools as part of the curriculum, including health controls, counselling, workshops, clinical care and contraception initiation, among others. An appointment is scheduled for the YFHS in the primary health centre only for interventions that need a clinical environment, such as LARC placements.
Type of healthcare model	Model that perpetuates the Health Centre as the central space for sexual and reproductive health care for young people. Although the relationship with schools is planned and frequent, it does not question the health model centred in an exclusive territory but reinforces it. Hand‐in‐hand with this, sexual and reproductive health is conceptualized as an isolated dimension of health.	Model that breaks with the sectoral logic of health and opens the territory, relating to the adolescent community on an ongoing basis. The model overcomes the conceptualization of sexual and reproductive health as a specific dimension of health by integrating it into a more holistic care model within the life context of adolescents.

LARC, Long Acting Reversible Contraception; YFHS, Youth‐Friendly Health Services

### 3.3 Strategies: Responses to adolescents’ sexual and reproductive health needs

In the “in‐and‐out” strategy, health professionals are located primarily in the health centres and move to schools to carry out specific activities. A midwife described their activities as follows: “We go to one school to do prevention week, in another we do workshops, in another we carry out health controls, and there we do education, prevention of pregnancy and sexually transmitted diseases.” Participant observation corroborated that these activities give accurate information about preventing sexually transmitted infections and pregnancy, mainly from a biomedical point of view. Nonetheless, they focus less on developing the affective dimensions of sexuality and the consequences of early parenthood in adolescents’ lives. While this model brings sexual and reproductive health information and counselling closer to adolescents, it perpetuates the idea that health is a topic situated mainly in its own territory, which is the health centre.

In the second strategy, “health rooms” or “welfare rooms” are implemented inside schools with health professionals continuously being present during school operating hours. According to the key informants, health professionals and adolescents who participated in the programme, this strategy has the following strengths:

1. Significant emphasis is placed on activities and workshops focussed on adolescent girls developing a life project, in order to reverse the idea of motherhood as a goal and an end in itself. As an example of the meanings of motherhood, one adolescent girl stated: “I wanted to become a mum because I wanted company, because I had my boyfriend but… it's not the same… a child is something that you'll always have, even if you leave home, it will always be your child, but the boyfriend can leave.” The programme seeks to motivate girls to finish school and continue with further studies and personal interests. A midwife expressed this idea powerfully: “So, the way [for adolescent girls] to validate themselves was to become pregnant, because ‘now I'm someone's mum, I have a responsibility’. But now that we're here, what we've done is to help change the mentality of these young people who didn't have a clear life project. (…) There we are, saying ‘I believe in you, I know that you can achieve your purposes’, and they understand that they can be validated in ways other than parenthood. That's really important.”
2. The health professionals who have been hired for working in schools have usually just graduated and have not previously worked in the health sector. They have therefore not been exposed to some of the public health sector's culture habits regarding working with adolescents, such as working in isolation from other sectors and reproducing prejudices towards adolescents (as a difficult age group that is not interested in health issues and constantly involved in risky behaviours). One of the key informants highlighted the importance of this measure: “The new professionals are hired specifically to work in schools, so they have never been part of the health centre. Taking them from the centres to go to work to schools would have been a disaster, because they have a particular culture, their own way of doing things. And it's very difficult to make them leave their working culture.”
3. Health professionals in schools are in constant contact with the adolescents, which enables them to establish affective bonds that facilitate their work in sexual and reproductive health and adolescent pregnancy prevention. Observation showed that when they are not in the welfare rooms with adolescents, they are walking around the school and using the public spaces, such as hallways, yards and the dining room, to establish informal conversations with adolescents. The interactions are caring and affectionate, with hugs, laughter, jokes and an informal tone of conversation. A 16‐year‐old female user of the programme commented: “Here there is trust (…). Here they give you confidence, they help you a lot, which I didn't expect. I expected the typical answer: ‘you have to take this prescription and go’, but here they welcome you, they help you, they ask, they worry, and they do many things (…). Then, it's like a real change, seriously it's a change, because the staff is kind and very loving.” One midwife reinforced this: “For adolescents, the bond is very important, it's no longer just giving young people counselling, talking to them about sexuality, but it's supporting them, showing affection for the adolescent. The bond that is established in this space is crucial.”
4. The constant presence of health professionals and lack of bureaucratic barriers mean that both young women and men can approach them. This tackles one of the health sector's biggest historical problems regarding adolescents: the invisibility and exclusion of young men's sexual and reproductive rights. One midwife addressed this issue: “In general the [Health] Ministry is focused on sexual and reproductive health in women, and men are left aside. In fact, now that we're in the school, young men go to the midwife and have that incentive. But if you ask in the street, for example, if they go to the midwife, it is like ‘wow, that's very difficult’. In fact, it's almost impossible to make them go to the youth‐friendly spaces. Men do go to the midwife in school, and we have the possibility, the opportunity to be there, but they don't go to the midwife in the health centre.” A 17‐year‐old young male user of the programme said: “I find it good because they open up to men, because it was assumed that it′s always women who have to take care of themselves [referring to contraception], but men also have to take care of themselves.”
5. Contraceptive methods are directly recommended by midwives in schools and any referral to the health centres to obtain and/or place contraceptive methods is quick since health professionals have access to the online data and can schedule appointments directly. As a nurse explained: “What adolescents are asking for is to initiate [contraceptive] methods in school. Girls only need to go to the welfare room, and there they are given a card, and with that card they go after school to the primary health centre, without having to wait in line, or having to make an appointment with the midwife. That makes life much easier for them.”

The continuous contact between health professionals and adolescents opens an opportunity to share their “worlds”, as one midwife explained: “That's why it is so powerful to be here… here with them [adolescents], each day things happen, stories. Yesterday I found a girl crying, ‘I can′t continue any more’, she was crying in the yard and… suicidal ideation, cut arms. I mean, there are situations every day, because it's the adolescent world, and in our case, it's the adolescent world with the highest level of vulnerability.”

At the time the field work was being undertaken, this new strategy was encountering some resistance deriving from the lack of an intersectoral culture linking health and education. There had been problems with identifying and regulating the roles and functions of health professionals within schools, and a certain amount of resistance from school staff to adjusting to the new scenario and recognizing the importance of the work carried out by their health peers. As a psychologist that is part of the programme stated: “It's hard for them [teachers] to visualize how important this is (…), the teacher sometimes doesn't let you take the child because he thinks it's not important.” Despite this, most of the interviewees were enthusiastic that the strategy was moving in the right direction.

## 4 DISCUSSION

Two strategies for intersectoral collaboration between health and education for the provision of sexual and reproductive health services for adolescents in Chile's Metropolitan Region have been described. While both strategies bring sexual and reproductive health services closer to adolescents, the findings suggest that the school‐based strategy responds better to adolescents’ sexual and reproductive health‐related needs, with access to contraception being fundamental, as suggested by literature.^38^ This strategy is overcoming many of the reported barriers to preventing adolescent pregnancy^16^, ^21^ because adolescents are able to continuously access information and care for sexual and reproductive health issues in their daily lives (school in this case), and they develop affectionate and trustworthy relations with care providers. This does not mean that care in the health centres lacks affection or trust, but it usually lacks the quality time and frequency needed to nurture caring relationships.

The school‐based strategy enables sexual and reproductive health to be understood as an integral dimension of adolescents’ lives, and it reinforces a holistic idea of health in which its components cannot be approached in isolation. It serves to strengthen the values of primary health, such as the right to the highest possible level of health, equity, solidarity and responding to the population's health needs.^39^ It also promotes a salutogenetic approach^40^ because, rather than focusing on a pathological and risk‐centred view of adolescents and adolescent pregnancy, it stimulates the health sector to link with the community and share the responsibility for healthcare. This facilitates the exercise of adolescents’ rights and wellbeing in the community, contributing as well to a healthier community as the risks associated with adolescent pregnancy decrease, such as the reproduction of poverty and gender inequalities.^2^, ^8^, ^9^

A technical consultation with key stakeholders about adolescent pregnancy in the Americas agreed on seven priority actions to accelerate progress on the topic: 1) make adolescent pregnancy, its drivers and impacts, and the most affected groups more visible with disaggregated data, qualitative reports and stories; 2) design interventions targeting the most vulnerable groups, ensuring the approaches are adapted to their realities and that they address their specific challenges; 3) engage and empower youth to contribute to the design, implementation and monitoring of strategic interventions; 4) abandon ineffective interventions and invest resources implementing proven ones; 5) strengthen intersectoral collaboration to effectively address the drivers of adolescent pregnancy in Latin America and the Caribbean; 6) move from boutique projects to large‐scale and sustainable programmes; 7) create an enabling environment for gender equality and adolescent sexual and reproductive health and rights.^10^

Our study contributes to actions 1) and 2) by adding information about specific interventions targeted at vulnerable adolescents from a qualitative approach. The World Health Organization has stressed the need to conduct research in various sociocultural contexts in order to identify feasible interventions that can be implemented on a large scale to reduce early pregnancy.^41^ This study seeks to generate recommendations for public health policies in Chile, thus contributing to actions 4), 5) and 6) by giving evidence of the strengths of certain intersectoral models of collaboration that could be scaled up. The study also contributes to an environment of greater gender equity and sexual and reproductive health rights, as stated in action 7). These actions are aligned with the UN Sustainable Development Goals (SDGs) and the Global Strategy for Women's, Children's and Adolescent Health where preventing adolescent pregnancy is seen as essential to improving adolescent survival rates, and also to preventing the marginalization and poverty of this group, and enhancing the societal contribution of girls and women.^10^ The findings of the study place adolescents in a position of vulnerability in terms of their sexual and reproductive health, and as their needs in this area become more visible, the urgency of working from participatory and rights‐based approaches becomes clear. In this context, we highlight as a central conclusion the urgency of promoting intersectoral strategies as a mechanism to promote adolescent's sexual and reproductive rights within an integral comprehension of health.

This study aimed to contribute to improving Chile's adolescent health policy through the following recommendations: (a) enhance the YFHS from an intersectoral perspective, seeking to expand the action area of the YFHS beyond health centres, promoting constant links with schools and communities; (b) give relevance to innovative strategies developed at the local level, translating them into operational guidelines that can be replicated elsewhere; (c) promote advocacy strategies between political decision‐makers and the education and health sectors, among others, to generate cross‐sectoral strategies and escalate them at the national level through an intersectoral policy on adolescent health. These recommendations can also be applied to other Latin American countries in which similar situations exist, given the sociocultural similarities that are shared in the region.

The authors seek to continue developing this line of research, exploring the development of territorial work in health beyond the educational sector, also considering the involvement of wider communities, which are considered strategic for the long‐term success of interventions in sexual and reproductive health.^42^, ^43^

The main limitation of the study derives from the inclusion of some municipalities of the Metropolitan Region of Chile, which make the results applicable to that Region and not generalizable to the population of the country. Nonetheless, the findings are consistent with other studies conducted in Latin America and Chile^3^, ^19^, ^24^, ^44^ and contribute to the limited information available on the access of adolescent men to SRH services.

## CONFLICT OF INTEREST

The authors have no conflict of interests to disclose.

## AUTHORS’ CONTRIBUTIONS

All authors have read and approved the manuscript being submitted, attest to the validity and legitimacy of the data and its interpretation. AO and MS participated in the design of the study, data collection and primary steps of analysis. All authors participated in the final steps of the analysis, drafting, revision and approval of the manuscript. We warrant that the article is an original work, has not received prior publication and is not under consideration for publication elsewhere.
